# Updating Ecological and Behavioral Aspects of the Sandfly Fauna in the Vale do Ribeira Region, São Paulo State, Brazil

**DOI:** 10.3390/insects12110988

**Published:** 2021-11-02

**Authors:** Byara Freitas Guedes Oliveira, Maria de Fátima Domingos, Fredy Galvis Ovallos, Vera Lucia Fonseca de Camargo-Neves

**Affiliations:** 1 Secretaria de Estado da Saúde de São Paulo, Superintendência de Controle de Endemias, Núcleo de Biologia e Comportamento de Vetores de São Vicente, São Vicente 11310-050, SP, Brazil; byara_guedes@hotmail.com (B.F.G.O.); fatimadomingos@uol.com.br (M.d.F.D.); 2 Faculdade de São Paulo—USP, Faculdade de Saúde Pública, Departamento de Epidemiologia, São Paulo 01246-904, SP, Brazil; galvisfregao@gmail.com; 3 Secretaria de Estado da Saúde de São Paulo, Superintendência de Controle de Endemias—Sucen, Departamento de Epidemiologia e Orientação Técnica, São Paulo 01027-000, SP, Brazil

**Keywords:** cutaneous leishmaniasis, sandfly, *Nyssomyia intermedia*, *Psathyromyia pascalei*, ecological index

## Abstract

**Simple Summary:**

Analyzing the biological and ecological characteristics of arthropods constitutes the basis for the entomological surveillance of vector-borne diseases. This is accomplished in order to implement vector surveillance and control programs. Thus, with the objective to update the distribution of sandflies in the main transmission region of cutaneous leishmaniasis in the state of São Paulo, we carried out a study of fauna in a modified environment, considering its environmental characteristics and climatic variables. Ecological indices such as richness, abundance, diversity, and equitability of the sandfly fauna in the region are presented.

**Abstract:**

Some ecological parameters and the distribution of vectors in the municipality of Eldorado, Vale do Ribeira Region, São Paulo, were studied. Entomological surveys were carried out from September 2019 to March 2021. It was observed that a few ecological parameters, including richness, abundance, diversity, and equitability, were typical of a modified environment, where artificial ecotopes maintain the presence of sandflies throughout the year. A total of 11,668 sandflies were captured. The presence of five taxa were observed in Eldorado, with low diversity and high dominance of *Nyssomyia intermedia* next to *Ny. neivai*, which are sympatric species. The results presented reinforce the importance of these species in anthropized areas in the transmission of cutaneous leishmaniasis (CL) agents and the need for entomological monitoring. *Psathyromyia pascalei* was encountered for the first time in the municipality, expanding the known area of distribution of this species in a modified environment.

## 1. Introduction

Cutaneous leishmaniasis (CL) is an infectious non-contagious chronic disease caused by several species of parasites in the genus *Leishmania* (Kinetoplastida, Trypanosomatidae). In Brazil, the most epidemiologically important species responsible for its dermotropic forms are *Leishmania* (*Viannia*) *braziliensis* (Vianna), *Leishmania* (*Viannia*) *guyanensis* (Floch), and *Leishmania* (*Leishmania*) amazonensis (Laison and Shaw). The clinical forms have different characteristics according to the infecting species, such as: cutaneous leishmaniasis (*L. guyanensis*), mucosal cutaneous leishmaniasis (*L. braziliensis*), diffuse cutaneous leishmaniasis (*L. amazonensis*), and disseminated leishmaniasis (*L. braziliensis*) [1]. The transmission of these parasites depends on the species of sandfly-vectors found in an area and distribution of the reservoir [1].

Sandflies are Diptera (Diptera, Psychodidae, Phlebotominae) whose females are hematophagous. Their average period of adulthood is 20 days, which is sufficient time for successful transmission of the protozoan. The species that have been identified as vectors of CL of the main etiological agent in the state of São Paulo (SSP), *Leishmania braziliensis*, are *Nyssomyia intermedia* (Lutz and Neiva), *Nyssomyia whitmani* (Antunes and Coutinho), *Nyssomyia neivai* (Pinto), *Migonemyia migonei* (França), *Pintomyia pessoai* (Coutinho and Barretto), and *Pintomyia fischeri* (Pinto) [2].

CL is widely distributed in the tropical and subtropical regions of the world, and Brazil has autochthonous cases in all Brazilian states. According to the Brazilian Ministry of Health (2020), in 2018, the national health notification system (Sinan) had 16,432 reported cases, with the mucosal form accounting for 5.6% of the cases, 6.7% in children under 10 years, with a detection rate of 7.8 cases per 100,000 inhabitants [3].

Between 2007 and 2019, the SSP contained 4577 registered autochthonous cases, with a detection coefficient of 1.0 case/100,000 inhabitants. The disease is a public health problem, given the high percentage of mucous forms that have been notified, which represent 21.4% of the total number of notified cases. The average for the period was 350 cases/year, of which 6.5% were recurring cases and 4.9% were cases in children under 10 years (SES-SP) (Source: SUCEN/CVE/CCD/SES-SP (2021)).

In the Vale do Ribeira, CL has been identified continuously since 1956 after the description of the first autochthonous cases by Forattini and Oliveira (1956) [4]. In the 1950s, after intense deforestation, CL almost disappeared in SSP, reappearing in the 1980s, with a significant increase in the disease [5]. The main vector of *Le. braziliensis* has been described as *Ny. intermedia sensu lato*, which has also been the most prevalent species in other regions of SSP [2,6,7,8]. The Vale do Ribeira region remains an important transmission area in the state, with the highest notification of CL cases in SSP (SES-SP). In the period from 1997 to 2019, 22.7% of the cases were reported with the place of infection in at least one of the 15 municipalities in the Vale do Ribeira region (SES-SP) (Camargo-Neves 2020, unpublished data). The municipality of Eldorado represented 15% of the reported cases in this region, thus highlighting its epidemiological importance (Camargo-Neves 2020, unpublished data) and no data reported of the sandfly fauna in this municipality. Whereas the sandfly fauna of the Vale do Ribeira was described as belonging to the *Ny. intermedia* complex, in this study we update the occurrence of sandfly species in an area of modified environment and ecological aspects of the fauna are analyzed.

## 2. Materials and Methods

**Figure 1:**
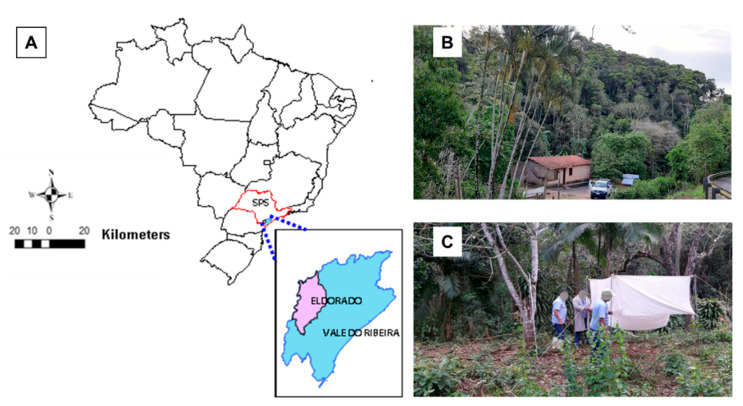
(**A**) Study area located in the municipality of Bairro André Lopes, Eldorado-SP, Vale do Ribeira region, São Paulo, Brazil; (**B**) collection point, aspects of peridomicile and, (**C**) capture site with Shannon trap.

Study area: Entomological surveys were carried out in the rural area of the municipality of Eldorado. This municipality is in the southern region of the ESP (−24.5020 S; −48.0849 W) in the Atlantic Forest biome, at an altitude of 64 m above sea level (Figure 1A). Average temperatures in recent years ranged from lows of 16.1 °C to highs of 25.2 °C (average annual variation of 7.4 °C). During this same period, the annual rainfall index was 1869 mm with a monthly average of 155 mm and annual relative humidity of 85% [9]. According to the last census carried out in 2010 [10], Eldorado has a population of 14,641 inhabitants, with 7205 inhabitants living in urban areas and 7436 (50.7%) in rural areas, indicating the population’s vulnerability considering they live in rural anthropized environments where the transmission cycle of CL occurs. With 1657 km^2^ and demographic density of 9.6 inhabitant per km^2^, the municipality is the fourth largest in territorial extension in the state of Sao Paulo. Economic activities include subsistence agriculture and livestock, in addition to plant extraction (banana culture) and tourism. Eldorado is one of the 29 municipalities in São Paulo considered a tourist resort [11,12].

The entomological captures were carried out in a residence located in a rural area called “pé do morro” (foothill), close to a forested area (Figure 1B). The peridomicile contained many domestic animals with the presence of dogs, chickens, and goats.

Entomological Collection: The entomological collections were conducted from September 2019 to March 2021 (except for the months of December/2019 and April/2020), for one night every fortnight. The collections were accomplished in a Shannon trap-type [13] (Figure 1C) tent operated by three catchers for an average period of three hours, starting 30 min after dusk.

The specimens were collected with the aid of a Castro’s aspirator or an electric aspirator (6V) and kept alive in entomological cages, where they were fed with a fructose or sucrose source, until their arrival at the Center for Biology and Behavior of Vectors in São Vicente, from the Endemic Control Superintendence, where they were killed, clarified, and identified according to Galati (2003) [14]. The initial and final temperature and humidity were recorded as well as the presence of animals during the capture period.

Data Analysis: Data were compiled in an Excel^®^ spreadsheet. The variables collected were date, capture time; initial and final temperature and humidity; presence of animals; number of catchers, and the registration of species by sex.

To estimate density, hourly effort per month was calculated, considering the number of catchers, and nights of capture.

The species’ richness, species’ accumulation rate, and statistical abundance analysis were estimated using the *EstimateS* version 9 statistical package [15]. Richness (S) was calculated as the number of species collected per survey night; the accumulation curve was calculated using the Coleman rarefaction method. The estimate of the true number of species was given by the estimator CHAO 1 and 95% confidence interval. Shannon diversity index (H), Simpson dominance index (λ), and Gini–Simpson equity index (1 − λ) using the statistical package PAST version 4.05 [16] were calculated by the average total number of captured specimens/hour/month for each species for the period in question. The Shannon diversity index was also calculated for each entomological survey, and a species’ accumulation curve was plotted. Spearman’s non-parametric correlation test [17] was used to correlate temperature and humidity with species; scatter plots were plotted in Excel^®^.

## 3. Results

In the study period, 11,668 sandflies were captured, of which 6369 were males. The male:female ratio observed was 1:1.25.

**Figure 2:**
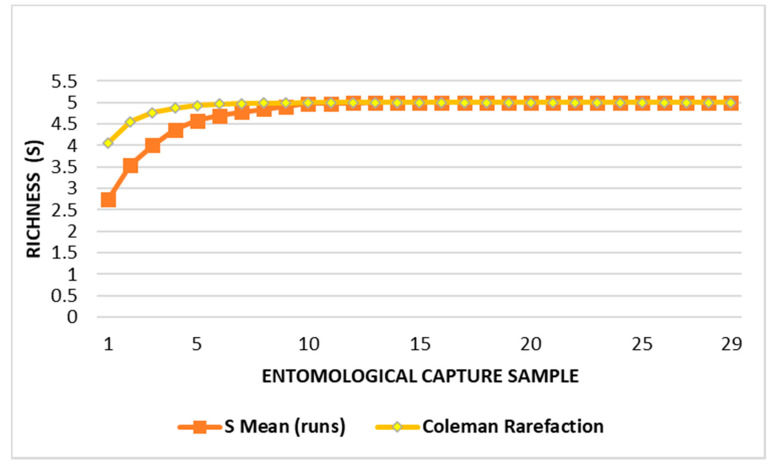
Species accumulation curve by the mean (S means) of the observed richness (S) compared to the expected number (Coleman rarefaction) in each collection.

The richness (S) was five taxa, with the resulting efficiency from the collection period equal to 100% (CHAO 1 = 5 [5.0 − 5.1]). Figure 2 charts the species accumulation curve considering all the captures conducted.

Of the specimens captured, 73.3% were identified as *Ny. intermedia* and 25.0% as *Ny. neivai* (Table 1). The following ecological indices were obtained: diversity H = 0.63; dominance λ = 0.60; and equity 1 − λ = 0.40.

**Figure 3:**
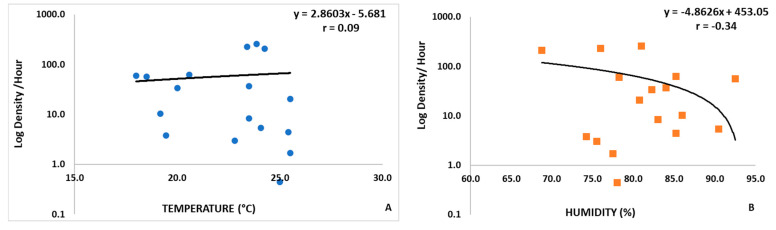
Dispersion graphs of the monthly hourly average for the total number of sandflies captured, monthly average air temperature (**A**), and relative humidity (**B**).

**Figure A1:**
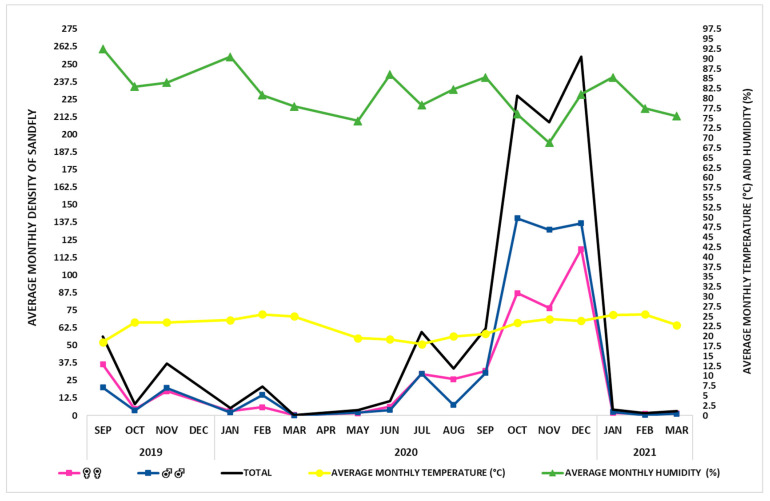
Monthly hourly average of sandflies captured and monthly average temperature and relative humidity. Eldorado, SP, September 2019 to March 2021. Note: In the months of December 2019 and April 2020 no surveys were carried out.

Their seasonality was noted in a prominent peak in 2020, starting in spring and ending in summer, correlated with relative humidity (average of 81.1%) and temperatures around 22.4 °C (Table 2). Although no correlation was observed between the temperature (r = 0.09) and relative humidity (r = −0.34) (Figure 3), there was an increase in the sandflies’ density with higher temperatures and a decrease with the rise of the relative humidity of the air (Figure 3, Figure A1). The total hourly average number of sandflies for a month for *Ny. intermedia* was 42.6 and 15.0 for *Ny. neivai*, with the highest values being observed in the spring for both years (Table 2, Figure A1).

## 4. Discussion

Some ecological parameters and the distribution of vectors in the municipality of Eldorado, Vale do Ribeira Region, São Paulo, were studied. In the study area *Ny. intermedia* s.s. is the predominant species, which represented 73.3% of the total specimens captured. The high density of *Ny. intermedia* reinforces the importance of this sandfly species as transmission agent of CL in the area, which has been described by several studies since the mid-1950s [18].

In a modified environment, *Ny. intermedia* s.l. was appointed as the main vector of *L. braziliensis* [18,19], mainly due to its high density in anthropized environments. Similar observations were described by Gomes et al. (1980) [20] and by Gomes and Galati (1989) [21], in studies on the adaptive capacity of *Ny. intermedia* s.l. in artificial ecotopes within the Vale do Ribeira region. However, Marcondes (1996) [22], based on morphological characteristics, observed the existence of two species, *Ny. intermedia* s.s. and *Ny. neivai,* so far considered as the same species. Therefore, data presented in this study contributes to the updating of information on these species in CL transmission foci in the state of São Paulo.

In the studied area, the modified environment and the presence of artificial ecotopes dominated by *Ny. intermedia* s.s. and closely followed by *Ny. neivai*, which are sympatric species in the coastal region of São Paulo, suggest that the adaptation of these species to the anthropic environment is unchanged. This has been previously observed in other municipalities of the Vale do Ribeira [8,20,21,23,24] as well as in other endemic areas of Brazil [25,26,27,28,29].

According to Andrade-Filho et al. (2006) [30], both species present a remarkable intraspecific and intrapopulational variation gradient; are morphologically very close with similar behavioral aspects, and are collected both in forests and in modified environments. The epidemiological importance of these two species is due to their known anthropophilic characteristic and the natural infection found by several studies [18,31,32,33,34,35,36].

The anthropophilic characteristic can also be attributed to *Ny. neivai*, given that the collections were conducted using a Shannon trap with humans as bait. In addition, its importance as a vector has been recorded through natural infection by *Leishmania* (*Viannia*) sp or *Leishmania braziliensis* [37,38,39,40]. This taxon, as well as *Ny. intermedia*, is adapted to the modified environment and can bridge the gap between the natural environment and the altered environment. Casanova et al. (2005) [41], in a marking, capture, and recapture study, found that the largest recapture of *Ny. neivai* females using Shannon traps exposed in wild and forest edge environments, demonstrating the tendency of this species to remain at the forest’s edge. However, infection is likely to occur when dispersing to the forest environment, as Shannon traps located in peri-domestic habitat support the hypothesis that household transmission results from infected females in an enzootic cycle which probably remain close to the human population [42,43,44].

Two other species of major epidemiological importance were captured. The *Migonemyia migonei* and *Pintomyia fischeri* species are known to be present in a modified environment and anthropophilic, as previously described in this region of the Vale do Ribeira in foci of cutaneous leishmaniasis [2,8,21,44]. These species are involved in the transmission of *L. braziliensis*, and its infection with this parasite has been registered in several endemic regions of Brazil [36,45,46,47,48]. Furthermore, *Mi. migonei* next to *Ny. intermedia* and *Pi. fischeri* have endophilic behavior, and can be infected outside the home and transmit the parasite to humans at home, contributing to the continuing transmission as observed in the municipality of Eldorado.

The finding of *Pi. fischeri*, even in low abundance, as observed in the studied area, is relevant due to its strong relationship with human habitat—its high anthropophily—which leads to this species being identified as a secondary vector of cutaneous leishmaniasis. In Eldorado, the high density observed of the more prevalent species, such as *Ny. intermedia* and *Ny neivai*, may be more favorable to CL transmission, to the detriment of *Pi. fischeri*, a fact corroborated by Galati et al., (2009) [49]. Nevertheless, its role as a secondary vector cannot be ruled out, since this species was registered with infection of *Leishmania (Vianna)* flagellated forms in Rio Grande do Sul [50] and its experimental infection has been proven [51]. Among the epidemiologically important vector species of CL, *Pi. fischeri* was the only one that was not naturally infected in SSP [52] but has been frequently found in high abundance in other areas of CL transmission [2,53].

Furthermore, *Mi. migonei* is a putative vector of *Leishmania infantum* [54] along with *Pi. fischeri*, whose natural infection was recorded by Pita-Ferreira (2005) [36] and experimentally its vectorial capacity demonstrated [55]. In addition, its high anthropophily and their joint distribution with the human cases of visceral leishmaniasis [56] point to the role of these two species of sandflies in the transmission of *L. (L). infantum* in the absence of *Lutzomyia longipalpis*. Therefore, the risk of visceral leishmaniasis transmission in the studied area should not be ruled out because the main vector is absent. The introduction of the infection is mainly through domestic dog, which is the main reservoir in the domestic environment and because of the cinophilic characteristic of these species.

The detection for the first time of *Psathyromyia pascalei* (Coutinho and Barreto) in the municipality of Eldorado is noteworthy even though this species has been observed in other municipalities in the Vale do Ribeira region [57,58]. So far, there is no evidence of its vectorial capacity for the transmission of *L. braziliensis* [59]. This encounter may reflect the proximity of the forest to the collection site, but also evidence of the domiciliation of the species, which deserves attention. Entomological monitoring should continue to verify future variations in the abundance of this species.

For the ecological indices, the low species diversity (H = 0.63) could be related to the degree of anthropization, and the existence of food sources (domestic animals) enhances the development of species close to the household, as described in the literature [25,27]. Deforestation in the Atlantic Forest region may have favored the adaptation of *Ny. intermedia*, *Ny neivai*, *Pi. fischeri*, and *Mi. migonei* [42,43]. In this study, the first two species showed dominance, representing 98.8% of the taxa captured (λ = 0.60), corroborating the statement that *Ny. intermedia* s.l. thrives in a modified environment at the expense of other species. Still considering its role as the main vector of CL in the state of São Paulo, a correlation was observed between the presence of CL cases and the presence of *Ny. intermedia* s.l., in 88% in the investigated municipalities [2].

The male:female ratio observed was 1:1.25, which was also an expected result for the environment modified by the capture method employed. Gomes et al. (1982) [24] observed that this behavior would be an endogenous mechanism of the species in which males and females would occur in the same proportion, including being captured during copulation, in captures carried out on the ground with human bait. As this behavior has not been observed in another municipality in the region [6], it was perhaps influenced by the capture method used.

The great plasticity of the phlebotomine population was observed throughout the year. The two most abundant species showed greater preference for the periods of the year, with an average relative humidity of 81,1% and average temperatures of 22.4 °C (Table 2). This behavior was also observed by Salomón et al. (2002) [60], in which *Ny. intermedia* showed peaks of exceptional abundance after the rains, during the prior season. However, its occurrence throughout the year can be favored by the ecotopes present in the peridomicile of the area. The same author [60] highlighted that the behavior of the species can be influenced by microecological characteristics caused by human activity, such as the presence of chicken coops in the study area. As these habitats of sandflies are characterized by small variations in temperature and humidity, the two species could be present throughout the year, notably *Ny. intermedia*.

Climatic factors, such as temperature, rainfall, and relative humidity have been related with the occurrence of sandflies [24,44,60,61,62,63,64]. Barata et al. (2004) [65] observed the interference of climatic factors on the density population of sandfly in an urban area of the municipality of Porteirinha (Minas Gerais, Brazil), indicating a significant correlation between the number of sandflies captured and rainfall and humidity. However, temperature had no significant effect on the dynamics of these insects in the region, as was observed in Eldorado, SP. Rutledge and Ellenwood (1975) [66] suggest that the seasonality of sandflies is related to the patterns of rainfall distribution that act by modifying breeding conditions. The highest density peak was evidenced between spring and summer in the year 2020, and was also observed in the same period in the previous year but at lower density (Table 2, Figure A1). This was probably due to the high relative humidity, which negatively interfered with the density of *Ny intermedia* and *Ny neivai.* The sandflies are commonly found in high densities during the hot and humid months and sometime in drier months [60,63,64]. This behavior (in hot and humid months) is characteristic of *Ny intermedia* sl in the Vale do Ribeira region [24,44]. Moreover, the preference of *Ny. neivai* has been described in hot and humid periods in other regions of SSP [27].

Long periods of drought or the rainy season are enough to change the dynamics of sandfly populations, even in these micro-habitats. Although seasonality studies should be conducted for at least two consecutive years to produce reliable and consistent data, reducing the margin of error provided by atypical years, it was only possible to carry out 17 months of capture fortnightly; however, we verified a plateau by the curve of species accumulation after the 9th week of capture (Figure 2) and the expected richness (S = 5) given by the CHAO 1 estimate was the same obtained in the study period. Furthermore, our results are consistent with those of other authors who studied this aspect in other municipalities of the Vale do Ribeira [8,24,49].

## 5. Conclusions

Five taxa were collected in the studied area in the Vale do Ribeira, and *Ny. intermedia* and *Ny. neivai* were the predominant species. The results reinforce the importance of these species in anthropophilic areas in the transmission of CL agents and emphasize the need for entomological surveillance with a goal to detect natural infection of sandflies, especially for the early detection of *Leishmania infantum*. The encounter of *Ps. pascalei* stands out, as this was the first time that it was found in the municipality, expanding the distribution area of this species in a modified environment. Future studies could focus on evaluating the circulation of the *Leishmania* species in those sandflies and evaluating blood feeding patterns.

## Figures and Tables

**Table 1:** Total sandflies captured and identified according to species and sex.

SPECIES	♀	♂	∑	%
*Nyssomyia intermedia*	3851	4633	8484	73.3
*Nyssomyia neivai*	1280	1676	2956	25.5
*Mygonemyia migonei*	61	27	88	0.8
*Pintomyia fischeri*	23	13	36	0.3
*Psathyromyia pascalei*	14	0	14	0.1
TOTAL	5229	6349	11578	100.0
Shannon index (H)	0.63			
Simpson index (λ)	0.60			
Gini–Simpson index (1 − λ)	0.40			

**Table 2:** Monthly hourly average of *Nyssomyia intermedia*, *Nyssomyia neivai* and the total number of sandflies captured, as well as, monthly average temperature and relative humidity.

Year	Month	Monthly Average				
		Temperature (°C)	Humidity (%)	*Nyssomyia intermedia*	*Nyssomyia neivai*	Sandfly Total
2019	SEP	18.5	92.5	36.9	19.2	56.4
	OCT	23.5	83.0	4.6	3.4	8.3
	NOV	23.5	84.0	24.6	9.2	37.0
	DEC	-	-	-	-	-
2020	JAN	24.1	90.5	2.9	0.2	5.3
	FEB	25.5	80.8	16.2	4.1	20.6
	MAR	25.0	78.0	0.4	0.0	0.4
	APR	-	-	-	-	-
	MAY	19.5	74.3	3.4	0.4	3.8
	JUN	19.2	86.0	7.6	2.6	10.3
	JUL	18.0	78.3	51.7	6.6	59.4
	AUG	20.0	82.3	28.9	4.4	33.4
	SEP	20.6	85.3	44.4	17.3	62.1
	OCT	23.4	76.0	160.3	62.3	227.5
	NOV	24.3	68.8	157.7	49.3	208.5
	DEC	23.9	81.0	178.9	72.2	255.3
2021	JAN	25.4	85.3	2.4	2.1	4.4
	FEB	25.5	77.5	1.1	0.6	1.7
	MAR	22.8	75.5	3.0	1.2	3.0
Average total		22.4	81.1	42.6	15.0	58.7
Minimum and Maximum values		(18.0–25.5)	(68.8–92.5)	(0.4–178.9)	(0.0–72.2)	(0.4–255.3)

Note: For the months of December 2019 and April 2020 no surveys were carried out.

## Data Availability

Not applicable.
